# Targeting Acidic Diseased Tissues by pH-Triggered Membrane-Associated Peptide Folding

**DOI:** 10.3389/fbioe.2020.00335

**Published:** 2020-04-28

**Authors:** Yana K. Reshetnyak, Anna Moshnikova, Oleg A. Andreev, Donald M. Engelman

**Affiliations:** ^1^Department of Physics, The University of Rhode Island, Kingston, RI, United States; ^2^Department of Molecular Biophysics and Biochemistry, Yale University, New Haven, CT, United States

**Keywords:** cancer, inflammation, imaging, therapy, pHLIP, fluorescence, PET

## Abstract

The advantages of targeted therapy have motivated many efforts to find distinguishing features between the molecular cell surface landscapes of diseased and normal cells. Typically, the features have been proteins, lipids or carbohydrates, but other approaches are emerging. In this discussion, we examine the use of cell surface acidity as a feature that can be exploited by using pH-sensitive peptide folding to target agents to diseased cell surfaces or cytoplasms.

## Introduction

Targeting therapeutic agents to diseased tissues can significantly enhance their efficacy by reducing side effects. Successful molecular targeting approaches have usually been based on distinguishing the cells in a diseased tissue from those in a healthy tissue by exploiting surface features. The advantages of targeted therapy have motivated searches for distinguishing features between the molecular cell surface landscapes of diseased and normal cells. In this discussion we emphasize an alternative for targeting: cell surface acidity.

Traditional approaches for targeting diagnostic and therapeutic molecules to the site of disease are based on recognition of overexpressed receptors or specific antigens localized at the surface or within cellular membranes (Even-Desrumeaux et al., 2011; Boonstra et al., 2016). However, variable expression of these markers presents difficulties, and, for example, in traditional targeting to treat tumors using molecular biomarkers, resistance by clonal selection often emerges, and compromises the therapy (Marusyk and Polyak, 2010; Gillies et al., 2012; Greaves and Maley, 2012). Moreover, cells in healthy tissues typically express the same proteins, reducing the specificity of targeting.

A very different set of targeting approaches might be based on the recognition of differences in other properties of the cellular membranes in normal and diseased tissues. It has been shown that cancer cells are mechanically softer than normal cells, and that the membranes of cancer cells have increased fluidity (Sherbet, 1989; Schierbaum et al., 2017; Rudzka et al., 2019). Further, bilayer asymmetry differs between normal and cancer cells, with phosphatidyl ethanolamine appearing in larger quantities in the outer monolayer (Tan et al., 2017). With abnormally high rates of cell division, cancer cells often exhibit such changes in membrane composition and asymmetry as they must upregulate biosynthetic pathways to provide cell building blocks, such as membrane components, rather than relying on circulating supplies. Membrane physical properties might potentially be utilized for selective targeting of diseased tissues and/or the cellular uptake of various delivery agents if means can be discovered to do so.

In addition to the targeting of biomarkers or membrane properties, cells within diseased tissues have pronounced surface acidity resulting from their metabolism. Such acidity can be specifically targeted by pH-triggered membrane-associated peptide folding, which is the subject of this review.

## Membrane-Associated Folding

The process of peptide insertion to span a membrane is of fundamental interest in evolution. It also illuminates our thinking about the ways that lipid interfacial regions can interact with the molecules they encounter (White et al., 2001). Membrane protein insertion and folding is facilitated by complex molecular machines *in vivo*, such as the translocon, which assist in placing transmembrane sequences across the bilayer (Cymer et al., 2015). Some moderately polar transmembrane domains can postranslationally translocate themselves into membranes. For example, C terminally anchored proteins, such as the apoptotic repressor Bcl-xL, can insert their C-terminal ends into membranes (Steel et al., 2002; Habib et al., 2003; Vargas-Uribe et al., 2013). Another example is the spontaneous insertion of diphtheria toxin triggered by low pH (Donovan et al., 1982; O’Keefe et al., 1992; Ladokhin, 2013; Vargas-Uribe et al., 2015). Studies of examples of spontaneous insertion and folding have inspired the design of synthetic peptides that are soluble in aqueous solution and spontaneously insert into membranes, and have been the subjects of biophysical investigations (Ladokhin and White, 2004). The discovery of peptides that respond to low pH by spontaneously inserting and folding across a membrane (Hunt et al., 1997) accelerated the development of these peptides for medical applications (Reshetnyak et al., 2006; Andreev et al., 2014; Wyatt et al., 2018a), as it emerged that low cell surface pH (or high acidity) is associated with several significant pathological conditions.

## Acidity in Diseased Tissues

An elevated level of extracellular acidity is found in tissues in pathological states such as cancer, inflammation (including neuro-inflammation), arthritis, stroke, ischemia, and others (Menkin and Warner, 1937; Reeh and Steen, 1996; Kedika et al., 2009; Pezzulo et al., 2012; Robbins and Swanson, 2014; Vidale et al., 2017; Pillai et al., 2019). In the case of ischemia and stroke, hypoxia and a compromised blood supply are primarily associated with the pathology; the cellular metabolism in these diseased tissues becomes partially anaerobic, leading to the production of acid from glycolysis (the Pasteur effect) (Krebs, 1972). An additional effect is found in malignant cancers and activated macrophages, which have an elevated uptake of glucose even with a normal oxygen supply, known as “aerobic glycolysis” or the Warburg effect (Warburg et al., 1927; Warburg, 1956). These cells continue to use glycolysis at a high rate, even when mitochondrial oxidative pathways are available, resulting in acidification that adds to any result of hypoxia (Swietach, 2019). Further, the production of carbon dioxide by the rapidly metabolizing cells causes expression of carbonic anhydrases on the cancer cell surfaces, promoting further acidification of the extracellular environment as the cells export the carbon dioxide and the anhydrases convert it into bicarbonate ions and protons (Wykoff et al., 2000; Potter and Harris, 2003; Swietach et al., 2009). Finally, the electrochemical potential across a cell membrane is positive on the outside of the cell, and will tend to concentrate hydrated protons and other cations near the surface.

The cytoplasmic production of acidity is deleterious, so cells have mechanisms to regulate their cytoplasmic pH by exporting the acidity to the extracellular environment. pH regulation is carried out by transmembrane proteins that pump protons from the cytoplasm across the plasma membrane to the extracellular space or to the lumen of various organelles (Damaghi et al., 2013). The flux of exported acidity lowers the pH surrounding a diseased cell, and the proton concentration is accentuated near the cell surface both by the flux and by the membrane electrochemical potential. As a result, the extracellular pH is lowest at the surfaces of diseased cells, where it is significantly lower than normal physiological pH and the bulk extracellular pH (Anderson et al., 2016). The low pH region persists at the cell surface even in well-perfused areas within diseased tissue (Gillies et al., 2012). The acidity on the surfaces of cells is a targetable characteristic that is not subject to clonal selection, and the level of acidity is a predictor of disease progression (Estrella et al., 2013).

## pHLIP^®^ Technology

### pH Triggered Insertion Into Membrane and Folding

pH (Low) Insertion Peptides (pHLIPs) utilize pH-triggered membrane insertion and folding to target acidic diseased tissues. The insertion can be used for the selective delivery of therapeutic and imaging agents (Wyatt et al., 2018a). pHLIPs constitute a large class of moderately hydrophobic membrane peptides that are soluble in aqueous solution at normal and high pHs. These peptides mainly consist of combinations of non-polar residues and negatively charged, protonatable residues (such as Asp and Glu, and their analogs) (Musial-Siwek et al., 2010; Weerakkody et al., 2013; Onyango et al., 2015). The presence of hydrophobic residues promotes adsorption to bilayer surfaces in most types of cellular membranes, and the adsorption is associated with a release of energy, mainly from hydrophobic interactions (Reshetnyak et al., 2008). pHLIPs containing fewer hydrophobic residues have lower affinities for membranes and much faster blood clearance (Weerakkody et al., 2013; Demoin et al., 2016). In addition, peptide adsorption to the membrane is modulated by membrane composition (Kyrychenko et al., 2015; Karabadzhak et al., 2018; Vasquez-Montes et al., 2018) and the local ionic environment (Schlebach, 2019; Vasquez-Montes et al., 2019; Westerfield et al., 2019). Significant attention has been given to the conformational states of pHLIPs adsorbed by membranes (Brown et al., 2014; Gupta et al., 2018; Vasquez-Montes et al., 2018). Such studies are challenged by the fact that pHLIPs do not adopt unique structures at the membrane surfaces, and a large variety of conformations is possible and dependent on individual pHLIP sequences (Wyatt et al., 2018b) or lipid compositions (Kyrychenko et al., 2015; Vasquez-Montes et al., 2018). No significant membrane distortion is created when pHLIPs partition into a bilayer outer leaflet in their non-helical unstructured forms (Narayanan et al., 2016), in contrast to amphiphilic pore-forming peptides, which partition into one leaflet of a bilayer as rigid alpha-helicies, inducing significant membrane tension and promoting membrane destabilization (Kornmueller et al., 2016; Masuda et al., 2019).

When the extracellular pH is low, key Asp/Glu residues are protonated, and the overall hydrophobicity (LogP) of the peptide is enhanced. Because the dielectric environment affects the protonation and deprotonation rates, the pKas of the carboxyl groups are raised in the environment of the membrane surface (Harris and Turner, 2002), usefully shifting them to physiologically relevant values near 6.0. Protonaton shifts the equilibrium and promotes peptide partitioning more deeply into the bilayer, which in turn triggers a coil-helix transition (folding) as the peptide backbone finds itself in a lower dielectric environment, favoring the systematic formation of intrahelical hydrogen bonds and a helical conformation (Popot and Engelman, 2000). The process is reversible: if the pH is raised, the exit of the peptide from the membrane induces unfolding (Andreev et al., 2010; Karabadzhak et al., 2012). The formation of helical structure at the surface of the membrane leads to membrane bilayer perturbation that promotes transmembrane insertion, relaxing the tension as the peptide adopts a stable transmembrane helical orientation. Kinetics studies (Andreev et al., 2010; Karabadzhak et al., 2012) and constant-pH molecular dynamics simulations (Vila-Vicosa et al., 2018) have provided insights into the peptide insertion and exit pathways. Thus, there is a reasonable set of concepts underlying the mechanism of pH sensing and insertion.

While there may be some agreement concerning the overall concepts discussed above, there is an area in which we believe there is an important conceptual misunderstanding found in the literature in the interpretation of protonation events, polypeptide partitioning and folding in a membrane and the targeting and intracellular delivery within a biological system. We assert:

• At each pH, an ensemble of all pHLIP states exists. At high and low pHs the predominant, but still not unique, states are the peptide membrane-adsorbed, and membrane-inserted states, respectively, each of which has a variety of dynamic excursions but is approximately described by the two states (Reshetnyak et al., 2007). At intermediate pHs a more distributed mixture of states is present. Therefore, any assumption of a sequential progression of intermediates from the membrane-adsorbed to the membrane-inserted state (Otieno et al., 2018) is a simplified view, which does not reflect the complexity of thermodynamic ensembles in real physical systems.
• The shift of the equilibrium from one distribution of states to another is triggered by pH changes in the environment near the lipid bilayer: a drop of pH induces protonation of Asp/Glu residues and the C-terminus. The pKa of protonation of each individual COO^–^ groups is dependent on the electrostatic potential, which varies dynamically with the ion concentrations, membrane composition and peptide insertion depth, as well as the presence of other groups dictated by the local dynamics of the peptide. Therefore, the pKa of any individual group is constantly changing in both directions while the average of the pHLIP ensemble of states propagates more deeply into the membrane (Vila-Vicosa et al., 2018). No fixed value of pKa can be assigned to any individual Asp/Glu residue or C-terminus during the course of insertion (Scott et al., 2017).
• The presence of positive charges at the peptide N-terminus and/or Arg residue at the N-terminal part, which are energetically costly to partition across a membrane barrier (Vila-Vicosa et al., 2018), predetermines the probable directionality of pHLIP insertion into the membrane, placing C-terminal end in, and the direction of the exit into the extracellular space (Karabadzhak et al., 2012).
• Since the insertion kinetics show timescales of ms to sec for the process (Karabadzhak et al., 2012), it is difficult to know which lipid bilayer organizational changes may accompany the insertion. Accurate dynamic modeling is problematical on this timescale, and results (Deng et al., 2013; Kong et al., 2014) should be regarded with caution.
• As noted above, in a biological system having living cells in acidic diseased tissues, a pH gradient exists in the vicinity of the cell membrane: at the cell surface the pH can be around 6.0 and it increases with distance from the cell surface (Stock et al., 2007; Anderson et al., 2016; Wei et al., 2019). Thus, the bulk extracellular pH is higher than the cell surface pH. The cell surface pH is relatively independent of tissue perfusion and will be low in both poorly and well perfused areas. Since pHLIPs sense pH at cell surfaces, where it is the lowest, an increase in the population of the membrane-inserted state is promoted.
• Living cells maintain electrochemical potential gradients across their membranes with the positive charge on the outside surfaces. The intracellular pH is about 7.3 in both healthy and diseased cells (Swietach, 2019), so the part of the potential that arises from a pH difference reverses as the external pH of a normal cell (∼7.4) is changed to ∼6.0. The Asp/Glu residues and the C-terminus at the membrane-inserted end of pHLIPs will be protonated as they insert across the membrane, but once in the cytoplasm they will be largely deprotonated in the cytoplasm. The resulting charges lead to an “anchoring effect,” in which the rate of the translocation of the charged COO^–^ groups back across the membrane is orders of magnitude lower compared to the rate of translocation in their neutral, non-charged COOH form (Kornmueller et al., 2016). Thus, there is a bias toward accumulation of the membrane-inserted state.
• Thermodynamic equilibration is not limiting either for pHLIP insertion into membranes or for the intracellular delivery of many polar cargoes. Given enough time for equilibration, even polar or charged cargoes will be delivered into cells and trapped in the cytoplasm if they are released from a pHLIP. However, in a living biological system there are limiting factors that come into play, including the circulation of the external medium, the normal turnover of the membrane via endocytosis, changes during growth and cell division, etc., so there are limits on cargo delivery based on the kinetic requirements of the insertion process, which may be very slow. Thus, the kinetics of insertion becomes a key factor for targeting and cargo delivery.

### Targeting and Extracellular Delivery of Cargo Molecules

**FIGURE 1:**
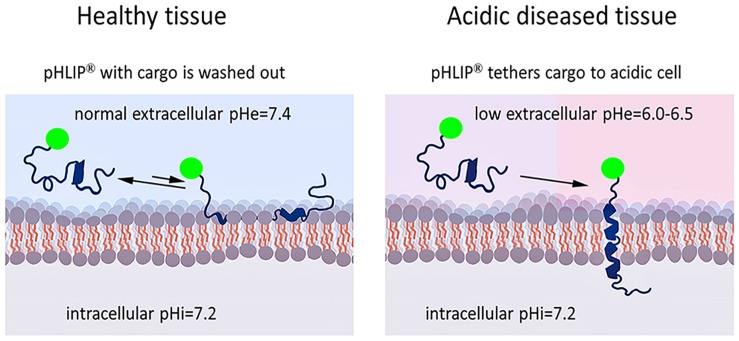
Targeted pHLIP delivery of cargoes to acidic cell surfaces. The cargo-pHLIP construct does not accumulate in healthy tissue cells with normal cell-surface pH (pH = 7.4) (left), since the pHLIP does not insert. The low pH (6.0–6.5) at the surface of a diseased cell causes pHLIP to insert across lipid bilayer to form a stable transmembrane helix, positioning the cargo at the surface (right). Examples of useful cargoes include imaging and immuno-stimulating agents, such as fluorescent dyes or antigens.

Peptides of the pHLIP family are members of the class of membrane-inserting peptides: they insert across lipid bilayers, leaving one terminus in the extracellular space and translocating the other one into the cytoplasm (Andreev et al., 2007; Thevenin et al., 2009). A variety of cargo molecules might be attached to the membrane non-inserting end to target them to acidic cell surfaces (Figure 1). As might be expected, there are few constraints on which cargoes can be targeted in this way. Examples of useful cargoes include imaging and immuno-stimulating agents. In addition to the delivery of small molecules, pHLIPs can direct a variety of nanoparticles and nanomaterials to the site of disease, which has been the subject of numerous publications including review articles (Han et al., 2013; Pereira et al., 2015), but is beyond the scope of this review.

Imaging agents coupled to pHLIPs have been used in animal models to label primary tumors, metastatic lesions and other acidic pathological states (Andreev et al., 2007; Reshetnyak et al., 2011; Daumar et al., 2012; Macholl et al., 2012; Sosunov et al., 2013; Weerakkody et al., 2013; Adochite et al., 2014; Cruz-Monserrate et al., 2014; Tapmeier et al., 2015). pHLIP-based PET (positron emission tomography) and fluorescent imaging agents are now on their way to human clinical trials. ^18^F-NO2A-pHLIP is a PET agent for imaging tissue acidity (Demoin et al., 2016), which will be administrated to breast cancer patients in a phase I clinical trial, and other PET isotopes such as 64Cu and 89Zr, might be used if longer half-lives would be desirable. The PET-pHLIP agents are expected to have applications in the imaging of a variety of tumors, and might also potentially prove to be useful for the diagnosis of brain tumors, where FDG is challenging to employ due to the high uptake of glucose by the normal brain. Applications in the imaging of other acidic diseased states, such as severe inflammation, atherosclerotic plaques, or arthritis, might also be developed. A PET-pHLIP agent potentially could have utility in the clinic for the recruitment of patients for treatment and to follow the effects of cancer therapy in general, and especially, immunotherapy. For example, the function of T-cells is inhibited by low pH (Huber et al., 2017) and potentially, immuno-therapy outcomes might be predicted, modeled and monitored using a PET-pHLIP imaging agent.

In another imaging application, a fluorescent agent has been developed using ICG, an indocyanine near infrared (NIR) fluorescent dye widely used for circulatory imaging (Desmettre et al., 2000; Alander et al., 2012). ICG-pHLIP is expected to be given as a single intravenous injection for tumor targeting and identification of margins and micrometastasis in the vicinity of primary tumors to improve surgical resections (Golijanin et al., 2016; Brito et al., 2019). To target and visualize tumors, imaging will be performed 24 h after IV injection of ICG-pHLIP to ensure blood and tissue clearance of the agent that is not bound in tumors, optimizing the contrast for marking cancerous lesions. It is important to note that the fluorescence is enhanced about 15–16 times compared to the emission in solution when pHLIP positions the ICG next to the membrane lipid bilayer (Golijanin et al., 2016; Roberts et al., 2019), thus providing additional enhancement of the tumor to background ratio. ICG-pHLIP can be easily adopted in clinical practice, since many clinical imaging systems for recording of ICG fluorescence are developed and currently in use in hospitals (Av et al., 2016; Nagaya et al., 2017).

Since the ICG-pHLIP resides in the blood for hours, imaging applications for angiography may also be useful. ICG-pHLIP interacts with blood proteins and remains circulating in the blood, allowing excellent visualization of blood vessels for a period of time of 1–2 h as compared to minutes for ICG alone. Blood flow imaging has wide ranging applications in a variety of surgical procedures (Desmettre et al., 2000; Alander et al., 2012), and has motivated the development of the imaging systems noted above.

A different, promising imaging agent is QC1-pHLIP (Roberts et al., 2019), where QC1 is a quencher molecule (as opposed to ICG, which is an emitter). QC1 is designed to effectively absorb light and transfer it into heat, which makes it an excellent agent for photo-acoustic imaging. Photo-acoustic imaging is a rapidly developing imaging modality (Valluru et al., 2016; Schellenberg and Hunt, 2018) and QC1-pHLIP could be a promising candidate for clinical translation for pre-operative imaging.

Animal studies have also demonstrated the potential utility of pHLIP linked to paramagnetic and superparamagnetic iron oxide nanoparticles for MRI, chitosan capped mesoporous silica coated gold nanorods and gold nanostars for computed tomography, photoacoustic imaging, and photothermal therapy (Janic et al., 2016; Zeiderman et al., 2016; Tian et al., 2017; Wei et al., 2017).

Importantly, in addition to the extracellular delivery of imaging agents, a variety of other cargoes, such as immuno-stimulating molecules including proteins (cytokines), carbohydrates, peptides (like HA-peptide), or small molecule antigens [such as 2,4-dinitrophenyl (DNP) and others] can be specifically targeted to the surfaces of tumor cells to induce biological responses. Specific antigens to activate either endogenous or exogeneous antibodies could be positioned on tumor cell surfaces by pHLIP, bypassing the need to identify molecular biomarkers for targeting. Endogenous antibodies could be developed against the targeted antigen by immunization. Or, exogenous antibodies or antibody drug conjugates (ADCs) could be administrated to target specific antigens delivered to tumor cells by pHLIP.

### Targeting and Intracellular Delivery of Cargo Molecules

**FIGURE 2:**
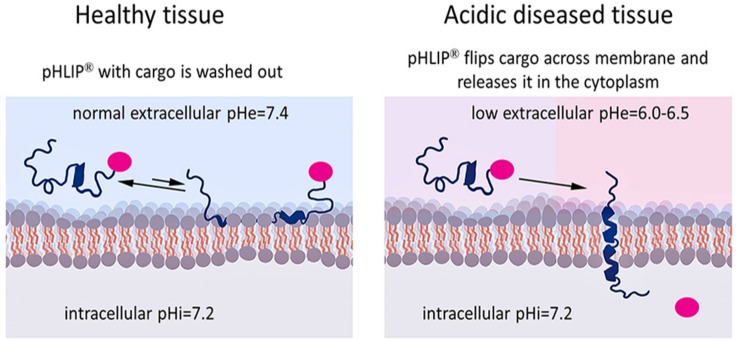
Targeted pHLIP delivery of cargoes to the cytoplasm. The pHLIP-cargo construct does not insert into cells with normal surface pH (pH = 7.4), but engages in transient binding that samples the surface pH and is then washed away (left). If the cell-surface pH is low (pH∼6.0–6.5), pHLIP samples the pH and inserts across the lipid bilayer, translocating the cargo directly into the cytoplasm (right). By using a link that is stable in the blood but cleavable in the cytoplasm, the cargo can be released inside the cell.

The pHLIP technology can also be used for the targeted intracellular delivery of molecules by connecting them to the inserting end of the peptide by linkers cleavable in cytoplasm, such as S-S links (Figure 2). If the cargo needs to be released in the cytoplasm in its original, non-modified form (as in the case of some small molecules) to prevent losing affinity to its intracellular target, self-immolating linkers could be used. Self-immolative elimination is a spontaneous and irreversible disassembly of a multicomponent compound into its constituent fragments through a cascade of electronic elimination processes. If a cargo can be released in cytoplasm in slightly modified form (as in the case of PNA or amanitin cargoes) a simple S-S linker (for example, SPDP crosslinker) can be used. It is important to note that the linker or additional modulators can facilitate the intracellular delivery of cargoes by lowering the effective membrane barrier (An et al., 2010; Wijesinghe et al., 2011; Moshnikova et al., 2013). If a cargo is polar, the hydrophobic linker/modulator may increase the LogP of the cargo-modulator. If the cargo is hydrophobic, a polar linker/modulator may decrease the LogP of the cargo-modulator to restrict off-target insertion.

**FIGURE 3:**
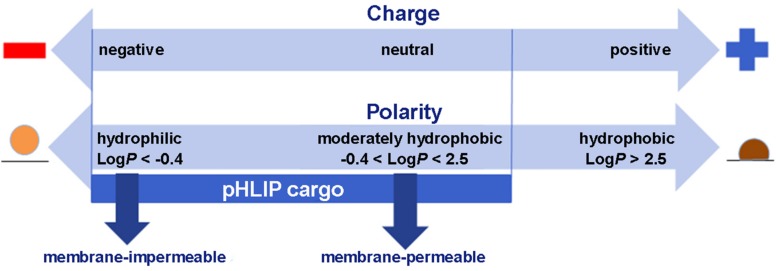
Some of the chemical properties of cargo molecules that can be targeted and delivered into cells by pHLIP. Two scales are shown, one representing the charges on a cargo and the other the Log of the Octanol-Water partition coefficient (LogP). Delivery by pHLIP is feasible for large, polar molecules that do not enter cells by themselves, expanding the range of pharmacology beyond that normally expected for drugs and attenuating leakage of the cargo from the cell after release. Some negative charge is acceptable, but positive charges slow the kinetics significantly. Moderately polar drugs can also be targeted, but very hydrophobic molecules disturb the insertion selectivity of pHLIP by adding hydrophobic membrane interactions and facilitating insertion at less acidic pHs.

Membrane-associated folding facilitates the cooperative translocation (flipping) of cargoes across the membrane bilayer directly into the cytoplasm, bypassing endocytotic uptake (Reshetnyak et al., 2006; An et al., 2010; Wijesinghe et al., 2011; Moshnikova et al., 2013). This pathway significantly expands the practical range of the molecular properties that can be used in pharmacological agents. Figure 3 summarizes the properties of cargo molecules best suited for pHLIP intracellular delivery. These range from polar or negatively charged cell-impermeable molecules to moderately hydrophobic molecules, including molecular weights from a few hundred Daltons to several kDa. The possible use of polar therapeutic cargoes presents a significant advantage for reduction off targeting and toxicity, since such molecules will not be able to enter cells on their own, and, once delivered, they will not readily exit the cell being targeted.

Peptide nucleic acids (PNAs) belong to such a class of useful, cell-impermeable polar molecules, which can activate immune responses and regulate cell functions by targeting coding and non-coding RNAs and DNA (Nielsen et al., 1991). PNA was one of the first polar molecules translocated into cells by pHLIP (Reshetnyak et al., 2006), followed by examples demonstrating *in vivo* targeting of miRNAs, long non-cording RNAs, and mRNAs (Cheng et al., 2015; Ozes et al., 2017; Zhao et al., 2018; Price et al., 2019; Sahraei et al., 2019). An especially attractive idea is to deliver a PNA to downregulate miR-21, which is the most commonly upregulated miRNA in solid tumors, and is associated with tumor pathogenesis during all stages of carcinogenesis. Tumor targeted intracellular pHLIP-mediated delivery of PNA targeting miR-21 in cancer cells and TAMs promotes an antitumoral immune response characterized by a macrophage-mediated improvement of cytotoxic T cell responses through the induction of cytokines and chemokines including IL12 and CXCL10 (Sahraei et al., 2019). Thus, the pHLIP-PNA immuno-stimulating effect allows the conversion of “cold” tumors into “hot” tumors, and the stimulation might enhance the therapeutic benefit of immuno-therapies that have already been developed. In addition to PNAs, a variety of immune-stimulating molecules, especially polar ones, like STING agonists, might be very good candidates for pHLIP intracellular delivery. In another application, the pHLIP-mediated delivery of PNA to target miR-33 prevented the formation of fibrosis in the kidney (Price et al., 2019).

Targeted pHLIP-mediated delivery of moderately hydrophobic small molecule drugs has proven to be successful as well. Among the drugs that have been delivered are potent inhibitors of tubulin, such as monomethyl auristatin E, poly (ADP-ribose) polymerase inhibitors (PARPi’s) including rucaparib and talazoparib, and other molecules (Burns et al., 2015, 2017; Song et al., 2016). A notable property is that pHLIP delivery has been shown to reduce bone marrow accumulation and toxicity, which is a significant issue in the use of many potent cytotoxic molecules. Thus, it appears that pHLIP delivery can reduce off-targeting, widen the therapeutic window and enhance the therapeutic index, which opens an opportunity to reconsider the use of very potent APIs for treatment of aggressive and metastatic cancers.

Other classes of potent therapeutic small molecules, such as the corticosteroids widely used in the treatment of severe inflammations and infections, might be targeted to restrict their action to the site of disease. Corticosteroids are very effective drugs that possess immunosuppressive properties. Dexamethasone is an example of a potent steroid with important clinical utility, but systemic administration and associated systemic immunosuppression are associated with devastating side effects, limiting the dose and duration of its uses (Johnson and Kelley, 2019). These limitations might be significantly reduced if targeted delivery could be used to treat inflamed tissues. pHLIPs can target inflamed tissues and fibrotic sites (Andreev et al., 2007), most probably by targeting activated macrophages.

### Features of the pHLIP Technology

To summarize, pHLIP peptides bind to the surface of tumor cells or cells in inflamed tissues, where the acidity is the most pronounced, followed by folding and insertion as helices across the membrane. Using the insertion of pHLIP, cargoes can be located at an acidic cell surface if the cargo is attached to the non-inserting end (extracellular delivery) and/or the cargo can be directly delivered and released into its cytoplasm if the cargo is attached to the membrane-inserting end via a bond that is unstable in the cytoplasm (intracellular delivery). The following advantages are associated with pHLIP targeted delivery:

• Cell-surface acidity and pHLIP targeting is not subject to clonal selection.
• pHLIP targeting overcomes the problem of antigen or other marker heterogeneity found within the tumor and between tumors.
• pHLIP tumor targeting can reach about 20% of the ID/g.
• pHLIP binding to a cell membrane is much less saturable than antigen binding, so larger amounts of cargo can be delivered.
• pHLIP can provide additional protection and increased stability of a drug in the blood by interaction with the ∼ 4 kDa pHLIP unstructured polymer.
• pHLIP alters the pharmacokinetics and biodistribution of drugs.
• pHLIP reduces off targeting and toxicity, especially in bone marrow, and can target highly potent molecules to tumors to enhance their therapeutic index.
• pHLIP directly flips cargo into the cytoplasm bypassing endosomal trapping.
• pHLIP’s cargo can be polar and large, reducing escape from the targeted cell.

## Conclusion

pHLIP technology is now taking its first steps into human imaging clinical trials, which potentially will open an opportunity for imaging of acidic diseased tissues, improvement of surgical resections of tumors, and visualization of blood flow. The initiation of imaging trials should be closely followed by the first trials of therapeutic interventions, such as the targeted delivery of cytotoxic and immune-stimulating or immune-suppressive molecules.

## Author Contributions

All authors listed have made a substantial, direct and intellectual contribution to the work, and approved it for publication.

## Conflict of Interest

YR, OA, and DE are founders and shareholders of pHLIP, Inc. The remaining author declares that the research was conducted in the absence of any commercial or financial relationships that could be construed as a potential conflict of interest.
